# Feasibility of Mass Screening for Colorectal Cancer Using Fecal Immunochemical Test in Iran

**Published:** 2017-12-01

**Authors:** Farin Kamangar, Mahsa Mohebtash

**Affiliations:** 1 Department of Biology, School of Computer, Mathematical, and Natural Sciences, Morgan State University, Baltimore, MD, USA; 2 Digestive Oncology Research Center, Digestive Disease Research Institute, Tehran University of Medical Sciences, Tehran, Iran; 3 MedStar Union Memorial Hospital Cancer Center, Baltimore, MD, USA

In this issue of the *Journal*, Salimzadeh and colleagues report on the feasibility, uptake, and positive predictive value of the fecal immunochemical test (FIT) to screen for colorectal cancer (CRC) and adenomas in the general population of Iran.^1^ The study is well designed, and the results are intriguing. FIT was offered in primary healthcare settings by primary care staff, and the uptake was quite high (1002 of 1044, 96%); the large majority (99.4%) of the received stool samples were satisfactory for testing.

The results are potentially important because: CRC is the third most common cancer in Iran, with an estimated 7163 new cases and 4262 deaths annually^2,3^; colorectal adenomas, in particular advanced adenomas, are precursors of CRC^4^; and screening for CRC has been found to be very important in reducing both incidence and mortality of CRC, as evidenced by experience of the United States and other countries. The incidence of CRC in the United States has declined at a rate of 2.5–4.0% per year over the past 15 years,^5^ which has been largely attributed to screening.^6^ Can FIT be used as a primary screening tool for CRC and adenomas in primary healthcare centers of Iran? Will it lead to a substantial decrease in morbidity and mortality from CRC?

Primary health care centers of Iran cover the majority of the population, particularly in the rural areas. These centers have been instrumental in enhancing the health status of the nation. For example, infant mortality rates decreased from 154 per 1000 in 1964 to 26 per 1000 live births in 2004,^7^ which was largely attributed to the effectiveness of Iran’s primary health care in delivering clean water, vaccines, and other primary healthcare measures to the entire nation, even in remote villages. As a result, life expectancy substantially increased from 45 years in 1960 to 76 years in 2015,^8^ and the major burden of diseases in Iran is now due to non-communicable diseases, including circulatory diseases and cancer.^9,10^ One might argue that primary healthcare centers should now shift their focus to primary and secondary prevention of cardiovascular disease, cancer, stroke, and renal diseases. The centers can provide services such as blood pressure control,^11^ diabetes control, and cancer screening. Currently, a major trial is ongoing on a polypill, comprising hydrochlorothiazide, aspirin, atorvastatin and either enalapril or valsartan.^12^ If the results are positive, polypill may be offered in primary healthcare settings to individuals who have a 10% or higher chance of cardiovascular incidents in the next 10 years.

Prevention of cancer, however, may be more challenging. While cancer overall is relatively common, each type of cancer is relatively uncommon, which poses a major obstacle for screening. For example, among the first 6466 deaths in the Golestan Cohort Study, 2662 were due to circulatory diseases, 1295 due to all malignant diseases combined, but only 67 due to CRC (unpublished data). Therefore, to reduce the number of cancers meaningfully, many people have to be screened to detect a small number of precancerous lesions or early cancers. In Salimzadeh’s study, the researchers offered FIT to 1044 people, of whom 91 (9%) were found to be positive. Of these, 45 people underwent endoscopy, and polyps were found in only 7 people (5 adenomas and 3 advanced adenomas). No cancers were found. On a larger scale, if mass screening is offered to all Iranians aged 50 – 75 years in one year—approximately 12 000 000 people— and 9% have positive FIT, over one million colonoscopies should be conducted. Given the very limited number of trained endoscopists in Iran (approximately 300), the large majority of those with positive FIT test will not undergo colonoscopy, which may cause undue stress for these individuals. Therefore, alternative approaches can be considered, which include: continuing the existing opportunistic screening approach; combining FIT with other sensitive tests; mass screening using other modalities such as sigmoidoscopy or CT colonography; mass colonoscopy of certain age groups; or a combination of the above.

The existing opportunistic approaches for CRC screening, combined with increasing public awareness of people at risk and signs and symptoms of CRC, could continue. Risk stratification tools can be used to reserve endoscopic services only for those subjects who are at higher risk and most likely to benefit. Examples include those with a family history of CRC, subjects with a history of abdominal radiation, those who have had a renal transplant, and those with inflammatory bowel disease.

FIT may be combined with stool DNA test (Cologuard), which has higher sensitivity and lower specificity than FIT for CRC and advanced adenomas.^13^ However, it is more costly than FIT, and there are no clear guidelines on how to follow negative colonoscopy after a positive stool DNA test. This may cause unnecessary follow-up colonoscopies.^13^

Using sigmoidoscopy or CT colonography may require less preparation and higher compliance rates. In Salimzadeh’s study, although uptake of FIT was high, colonoscopy compliance rates among individuals with a positive FIT was 60.0%. As reflected in many guidelines, the best strategy to make CRC screening most effective is to take patient adherence and preference into account.^6,14^ One barrier could be the vigorous preparation and sedation that are required for colonoscopy. Flexible sigmoidoscopy every 10 years combined with annual FIT is one of the US Preventive Services Task force (USPSTF) recommendations for CRC screening.^15^ Flexible sigmoidoscopy does not require significant preparation or sedation and can be performed by non-gastroenterologist clinicians trained for this purpose. Sigmoidoscopy may be an attractive option also because a large percentage (over two-thirds in one study) of CRCs in Iran are leftsided.^16^ CT colonography is almost as sensitive as colonoscopy; it requires bowel preparation but not sedation. Positive results need to be followed by colonoscopy, but negative results can delay the need for colonoscopy.

Mass colonoscopy campaigns may be considered for certain age groups—for example, for those who reach 50 years of age—which is approximately one million people each year. Of these, around 90 000 will have positive FIT tests and 50 000 will choose to undergo colonoscopy. That will be almost 160 extra colonoscopies per endoscopist per year, a reasonable additional load. Furthermore, this approach allows for the training of more endoscopists over time. Perhaps a more prudent approach is to do colonoscopy on people with a higher incidence rate, i.e., those who reach 60 years of age, just a few years before the peak age of 65 for this cancer. That way, many adenomas and early cancers can be diagnosed.

In summary, the study by Salimzadeh and colleagues has shown that using FIT as an initial screening tool is feasible in primary healthcare settings in Iran. However, mass screening of the entire population may not be feasible due to limitations in the number of endoscopists, major financial burden, and stress caused by the results when follow-up may not be possible for all. Measured approaches need to be carefully discussed and planned to make sure that the system is capable of providing the needed services.
